# The Comparison of Dynamic Condylar Screw Plate to Proximal Femoral Nail in Reverse Oblique and Transverse Intertrochanteric Fractures: A Retrospective Study on 61 Patients

**DOI:** 10.7759/cureus.36397

**Published:** 2023-03-20

**Authors:** Ersin Şensöz, Selim Ergun, Mahmut Enes Kayaalp, Engin Eceviz

**Affiliations:** 1 Orthopedics and Traumatology, Dr. Lutfi Kirdar Kartal City Hospital, Istanbul, TUR

**Keywords:** reverse oblique, intramedullary, fracture fixation, open fracture reduction, hip fractures

## Abstract

Objective: Reverse oblique (RO) and transverse intertrochanteric fracture patterns constitute a challenge for the operating surgeon. Currently, no gold standard fixation method exists. This study aimed to retrospectively compare proximal femoral nail (PFN) to dynamic condylar screw (DCS) plating in the treatment of RO and transverse intertrochanteric fractures.

Methods: A total of 61 patients fixated by PFN or DCS were included. Of these, 36 were treated with PFN (21 females and 15 males; mean age: 65.52 years), and 25 were treated with DCS (12 females and 13 males; mean age: 59.36 years). The mean follow-up time was 33.8 and 42.6 months (range: 24-108). Radiological evaluation included the quality of fracture reduction, neck-shaft angle change, posteromedial support presence, and bone union time. Complications such as mechanical failure, nonunion, and infection were noted.

Results: The only significant differences between the fixation methods were the superiority of DCS over PFN in earlier fracture union time (mean values: 8.9 versus 14.1 weeks) and the superiority (p=0.007) of PFN in shorter hospital stay (3.4 days versus 5.1 days). No significant difference was observed in radiological parameters. While similar mechanical complication rates were found, a significantly higher nonunion rate was detected with the DCS.

Conclusion: The most crucial disadvantage of DCS was the high rate of nonunion. Closed fracture reduction in PFN seems to be the most critical parameter to prevent severe complications. The open reduction using DCS showed no advantages over closed reduction and PFN fixation in providing a more anatomical alignment in AO/Orthopaedic Trauma Association (OTA) 31-A3 fractures. However, we recommend PFN application in this type of fracture, since nonunion is more common in DCS.

## Introduction

A reverse oblique (RO) or transverse intertrochanteric fracture is an unstable fracture pattern. According to the AO/Orthopaedic Trauma Association (OTA) classification, these fractures are labelled as AO/OTA 31-A3. A3 fractures are further subdivided into A3.1, A3.2, or A3.3, as to the presence of simple oblique, simple transverse, or multi-fragmentary/wedge fracture pattern. In A3 fractures, the lateral cortex integrity is compromised, unlike most stable intertrochanteric fractures of the same region. Among all hip fractures, RO trochanteric fractures have a frequency of less than 2% [1]. The loss of the integrity of the lateral cortex is a challenge for orthopedic surgeons by limiting surgical fixation options and increasing the complication rate [1-4]. Dynamic hip screw (DHS) systems are not recommended for these fractures because the 135° lag screw does not cross the fracture line perpendicular to it, promoting fracture separation rather than compression [1].

In order to overcome this problem, a dynamic condylar screw (DCS) system with a 95° lag screw, initially designed for distal femur fractures, has been frequently preferred for the treatment of AO/OTA 31-A3 fractures [1,5,6]. However, screw cutout or implant failure is the reported mechanical complication [6,7]. To avoid these risks encountered with the DCS system, an intramedullary implant, proximal femoral nail (PFN), was introduced. Nail fixation was shown to theoretically provide better biomechanical properties in the fixation of these fractures [8]. However, inadequate/relative fracture reduction, secondary varus displacement, screw cutout Z-effect, and reverse Z-effect might be the undesirable consequences of the PFN system [9].

Although in vitro studies showing the biomechanical superiority of PFN in treating AO/OTA 31-A3 fractures are available in the literature [10,11], no consensus has yet been reached in addressing these unstable fractures [7,12].

This study aimed to retrospectively review patients with AO/OTA 31-A3 fractures who underwent internal fixation with PFN or DCS and compare them in terms of radiological outcomes and complication rates. It was hypothesized that DCS would provide superior radiological outcomes but would lead to more mechanical and clinical complications than PFN. The rationale was that the DCS system, with its plate placed on the lateral wall of the femur, would provide more anatomical fracture reduction and better compression in the lateral cortex; however, the necessity of open surgical reduction would lead to various complications.

## Materials and methods

Among the 101 AO/OTA 31-A3 fracture patients operated on between 2013 and 2018, only patients with a follow-up of more than two years and fixed with either the DCS system or the PFN system with two lag screws were included in the study. PFN fixation with open fracture reduction, concomitant lower extremity fracture, open fractures, pathological fractures, and PFN systems with a single lag screw were excluded. The study was conducted retrospectively on the remaining 61 patients.

**Figure 1:**
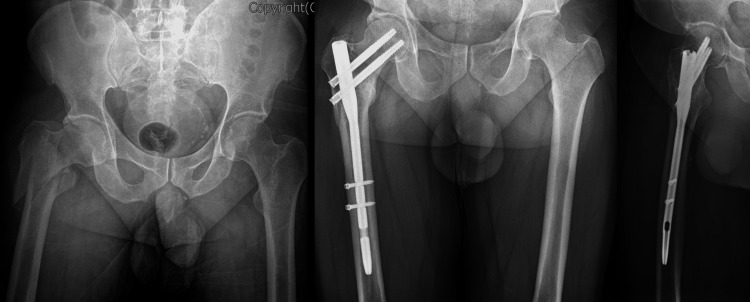
PFN fixation in reverse oblique hip fracture PFN: proximal femoral nail

Among the 61 patients, 36 were treated with the PFN, and 25 were treated with the DCS system. The mean follow-up period was 33.8 and 42.6 months (range: 24-108). All operations were performed on a traction table with the patient in a supine position and using C-arm fluoroscopy. All surgeries were performed by three senior orthopedic surgeons related to trauma surgery. If closed fracture reduction had been achieved, internal fixation with an intramedullary implant PROFIN PFN nail (TST®, Istanbul, Turkey) was inserted by a minimally invasive technique through the medial border of the greater trochanter (Figure 1). The nail has two non-locking 8.5 mm lag screws and one or two 5 mm distal locking screws. Patients with a cable or side plate placed through an additional incision were excluded.

**Figure 2:**
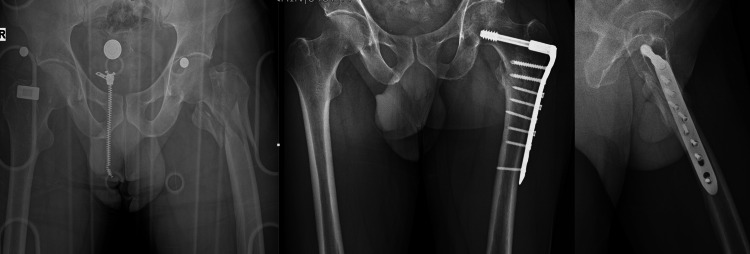
DCS fixation in reverse oblique hip fracture DCS: dynamic condylar screw

In our clinic, some surgeons prefer fixation with DCS. In patients operated on with DCS plating, a vastus lateralis split approach was used. Open fracture reduction was achieved, and the plate length was selected according to fracture extension (TST®, Istanbul, Turkey) (Figure 2).

Postoperative treatment included early mobilization and low-molecular-weight heparin prophylaxis for deep vein thrombosis for two weeks. Weight-bearing was not allowed before postoperative week 4, and gradual weight-bearing was initiated according to radiographic signs of callus formation. Patients were followed up in the outpatient clinic periodically.

Radiological evaluation was performed separately by two experienced orthopedic surgeons. These two surgeons performed independent radiological evaluations. They only came together at points where they thought differently and made a decision.

On the first postoperative radiographic view, the quality of fracture reduction was graded as good, acceptable (5-10° varus/valgus and/or ante- or retroversion), or poor (>10° varus/valgus and/or ante- or retroversion) [13], Besides the reduction quality, neck-shaft angle, the presence of posteromedial support, and bone union time (callus formation in at least three cortices [14]) were radiologically assessed. To not adversely affect the radiological evaluation, magnification was performed over the lag screw length [15]. While the quality of reduction, the presence of posteromedial support, and neck-shaft angle were evaluated in the first postoperative radiograph, the change in neck-shaft angle and bone union time was calculated according to the radiographs taken during the follow-up visits. Finally, the length of hospital stay and complications such as mechanical failure, nonunion, and deep infection that require surgical debridement were recorded.

Statistical analysis was performed using two tests. Student’s t-tests were used to compare the two groups with regard to mean age, mean follow-up duration, bone union time, and hospital stay. Fisher’s exact tests were performed to compare the groups with regard to gender, the side of injury, the energy of the trauma, AO/OTA classification, lateral butterfly fragment, body mass index (BMI), fracture reduction quality, posteromedial cortical continuity, neck-shaft angle change, and complications. A difference was considered to be statistically significant when p<0.05.

## Results

There was no significant difference between the groups in terms of mean age, gender distribution, affected side, and fracture type distribution.

Mechanical complications were detected in five patients (14%) with the PFN system (one lag screw cutout, one Z-effect, and three reverse Z-effect) and two plate breakage (8%) with the DCS system. The revision of the initial fixation was necessary for one patient with reverse Z-effect, one PFN lag screw cutout, and two DCS plate breakage. All revisions were done with a new DCS system. Three Z-effects and one reverse Z-effect patients were followed until the bone union was achieved and prominent lag screws were removed; however, revision surgery was not necessary.

Nonunion was only encountered with the DCS system in four patients (16%), which was significantly higher than the PFN system (p=0.02). One was revised with a PFN system, and the union was achieved; three were revised with hemiarthroplasty. Although the total number of patients who needed fixation revision was higher in the DCS system, there was no significant difference (Table 1). No significant risk factor was present for mechanical complications or nonunion regarding neck-shaft angle change, posteromedial cortical discontinuity, lateral butterfly fragment presence, and poor postoperative reduction quality.

**Table 1:** Comparison of the complication rates PFN, proximal femoral nail; DCS, dynamic condylar screw

	PFN (n=36)	DCS (n=25)	P value
Mechanical failure			
Implant breakage	0	2	
Lag screw cutout	1	0	
Z-effect/reverse Z-effect	4	0	
Total	5	2	0.51
Nonunion	0	4	
Infection	1	3	0.15
Revision surgery for fixation	2	6	0.035

One patient with PFN and three patients with the DCS system showed signs of deep infection regarding the infection (p=0.15). All resolved with early surgical debridement and intravenous antibiotic treatment, without a need for implant removal (Table 1).

## Discussion

The current study showed that patients with an AO/OTA 31-A3 fracture operated on with either DCS plating or PFN yield similar outcomes except for nonunion rates. While faster bone union was achieved with the DCS plating, a higher complication rate of nonunion (16%) and a longer hospital stay were shown as disadvantages. This may be related to open reduction. Contrary to our hypothesis, there was no significant difference between these two fixation methods in terms of the reduction of quality.

Mechanical complications such as varus malunion, lag screw cutout, and implant failure were found to be high [1], as A3 fractures are considered unstable due to the disruption of the lateral femoral cortical integrity [14]. Complications are even more likely, especially in cases where the lateral femoral cortex is fractured with a butterfly fragment or posteromedial cortical discontinuity is present following internal fixation [16-18]. Although the anatomical reduction of the fracture can theoretically be achieved more properly with open reduction and the use of a side plate in the DCS system, increased bleeding, surgical time, and infection rates might be listed as possible disadvantages [3]. On the other hand, PFN fixation, when performed with closed fracture reduction, is a minimally invasive surgery and biomechanically more stable than DCS plating [10]. Several authors proposed different techniques to overcome the difficulty in fracture reduction in these fractures. Kumar et al. reduced the fracture with the help of a plate and clamp [19]. Imerci et al. [20] and Bhat et al. [21] proposed to provide lateral cortex continuity by open reduction and with the help of cerclage wires. Lateral cortex continuity can be very important. Faster union in patients with DCS may be due to the support of DCS to the lateral cortex. However, all these techniques require open fracture reduction and therefore increase the risk of infection, devascularization of the bone, and possible nonunion. In this retrospective study, a significantly higher rate of nonunion was seen in patients operated on with DCS plating fixation compared to PFN. Moreover, similar radiological reduction quality was found in both fixation methods, contrary to the study hypothesis. An increased rate of infection in the DCS group that required surgical debridement and intravenous antibiotic treatment was detected, but the difference was nonsignificant (p=0.15).

The inability to achieve anatomical reduction is one of the most critical risk factors that increase failure in RO fractures [22,23]. According to the study by Turgut et al., varus malreduction and implant malposition are the most important known risk factors in this respect [24]. However, varus malreduction, posteromedial cortical discontinuity, lateral butterfly fragment, or poor postoperative reduction quality were not found as risk factors for the patients with mechanical complications and nonunion. Although open reduction was applied in patients who underwent DCS plating, anatomical reduction could not be achieved in all of them. This is one of the limitations of our study. The tip apex distance (TAD), which is another known risk factor for mechanical failure, can only be measured on patients with the PFN system in this study, not DCS [23,25,26].

There were only two studies that clinically compared DCS to PFN in AO/OTA 31-A3 fractures [7,12]. In a prospective study by Sadowski et al., operation time and hospital stay were shorter in the PFN group, and the failure rate was also significantly lower [7]. We also found shorter hospital stay in the PFN group in our study.

According to the study by Elis et al., there was almost no difference between PFN and DCS regarding operation time, hospital stay, malunion, and neck-shaft angle measurements, similar to the current study [12]. However, open fracture reduction was preferred in some patients undergoing PFN. In all the patients in the current study, who were operated with PFN, reduction was accomplished with minimally invasive techniques if necessary, without any open reduction.

This study had some limitations. First, the study was retrospective and thus susceptible to missing data and selection bias. Second, the sample size is relatively small due to the low incidence of A3 fractures but one of the highest reported in the literature. Third, we did not have any data on surgical time, bleeding amount, and bone mineral density of the patients. Lastly, although open reduction was applied in patients who underwent DCS plating, anatomical reduction could not be achieved in all of them. This might be related to the difficulty of open reduction in these fractures as reported previously.

## Conclusions

The higher complication rate of nonunion seems to be the most crucial disadvantage of DCS plating in AO-OTA 31-A3 fractures, although a shorter union time was seen in patients with a union in this group. Open reduction with DCS plating provided no advantage in obtaining a more anatomical alignment. Mechanical failure rates were nonsignificantly different between DCS plating and PFN. However, we recommend PFN application in this type of fracture, since nonunion is more common in DCS.
